# Cytotoxic Immunity in Peripheral Nerve Injury and Pain

**DOI:** 10.3389/fnins.2020.00142

**Published:** 2020-02-21

**Authors:** Alexander J. Davies, Simon Rinaldi, Michael Costigan, Seog Bae Oh

**Affiliations:** ^1^Nuffield Department of Clinical Neurosciences, John Radcliffe Hospital, University of Oxford, Oxford, United Kingdom; ^2^Department of Anesthesia, Boston Children’s Hospital, Harvard Medical School, Boston, MA, United States; ^3^Department of Neurobiology, Boston Children’s Hospital, Harvard Medical School, Boston, MA, United States; ^4^Department of Brain and Cognitive Sciences, College of Natural Sciences, Seoul National University, Seoul, South Korea; ^5^Dental Research Institute and Department of Neurobiology & Physiology, School of Dentistry, Seoul National University, Seoul, South Korea

**Keywords:** neuropathic pain, peripheral neuropathy, nerve injury, cellular cytotoxicity, innate immunity, natural killer cells, neuro-immunology, neuron-glia crosstalk

## Abstract

Cytotoxicity and consequent cell death pathways are a critical component of the immune response to infection, disease or injury. While numerous examples of inflammation causing neuronal sensitization and pain have been described, there is a growing appreciation of the role of cytotoxic immunity in response to painful nerve injury. In this review we highlight the functions of cytotoxic immune effector cells, focusing in particular on natural killer (NK) cells, and describe the consequent action of these cells in the injured nerve as well as other chronic pain conditions and peripheral neuropathies. We describe how targeted delivery of cytotoxic factors via the immune synapse operates alongside Wallerian degeneration to allow local axon degeneration in the absence of cell death and is well-placed to support the restoration of homeostasis within the nerve. We also summarize the evidence for the expression of endogenous ligands and receptors on injured nerve targets and infiltrating immune cells that facilitate direct neuro-immune interactions, as well as modulation of the surrounding immune milieu. A number of chronic pain and peripheral neuropathies appear comorbid with a loss of function of cellular cytotoxicity suggesting such mechanisms may actually help to resolve neuropathic pain. Thus while the immune response to peripheral nerve injury is a major driver of maladaptive pain, it is simultaneously capable of directing resolution of injury in part through the pathways of cellular cytotoxicity. Our growing knowledge in tuning immune function away from inflammation toward recovery from nerve injury therefore holds promise for interventions aimed at preventing the transition from acute to chronic pain.

## Introduction

The role played by the immune system in the context of nerve injury and pain is extensive (Ren and Dubner, 2010; Totsch and Sorge, 2017; Raoof et al., 2018). The two key effector mechanisms of the cellular immune response are inflammation and cytotoxicity. The importance of inflammation has long been recognized. The cardinal signs of inflammation – calor (warmth), dolor (pain), tumor (swelling), and rubor (redness and hyperemia) – were first documented by the Roman physician Celsus in the 1st century AD (Scott et al., 2004). These symptoms are in large part the product of the actions of inflammatory mediators such as chemokines, cytokines and vasodilators released from immune cells in a process designed to protect and facilitate the repair of injured tissue. The role of cytotoxicity, on the other hand, is to kill living cells and/or degenerate tissues via apoptotic mechanisms and is a critical function of immunity against cancer – another term introduced to the latin lexicon by Celsus (Hajdu, 2016). Pathways leading from immune activation to inflammation and their contribution to the generation of neuropathic pain are relatively well documented (Calvo et al., 2012). However, recent evidence points to a role of cytotoxicity in the immune response to nerve injury, which can in turn affect pain outcomes (Davies et al., 2019).

Neuropathic pain is defined as that arising from a lesion or disease of the somatosensory nervous system (Treede et al., 2008; Costigan et al., 2009b). Peripheral neuropathies leading to pain almost invariably result from a partial loss of innervation. This may occur either from disconnection of nerve branches from their peripheral target by physical injury (Bennett and Xie, 1988; Seltzer et al., 1990; Kim and Chung, 1992; Decosterd and Woolf, 2000) or a dying-back of axons from their terminals due to metabolic (O’Brien et al., 2014) or chemical neurotoxicity (Hoke and Ray, 2014). Axonal loss after injury is characterized by the mechanisms of Wallerian degeneration: a neuron-autonomous (i.e., self-determined) process involving active metabolic signaling within the axon leading to cytoskeletal destabilization and fragmentation (Gerdts et al., 2016; Llobet Rosell and Neukomm, 2019). Additionally, dying back of axons utilizes apoptotic pathways, while protecting the cell body from death (Yaron and Schuldiner, 2016).

The cellular response to nerve injury involves dedifferentiation of resident Schwann cells, and dynamic infiltration of systemic immune cells (Rotshenker, 2011). Normally this process would clear the way for axonal regeneration and nerve repair, resulting in transient hypersensitivity followed by recovery. However, interruption of this complex cascade of degeneration and regeneration can lead to aberrant processing which in some cases can transition into chronic pain (Xie et al., 2017; Davies et al., 2019). Thus, peripheral neuropathies lie at the intersection of multiple degeneration, immune and cell death-related pathways, and how these interact influences pain outcome over time.

## NK Cells as Surveyors of Stress and Disease

Natural killer cells are a key component of the innate immune response (Eberl et al., 2015), originally identified in the 1970’s as a population of large granular lymphocytes. NK cells were found to be spontaneously cytotoxic to freshly isolated cells and many tumor or immortalized cell lines (Timonen et al., 1981). Toxicity occurred despite the lymphocytes originating from healthy donors, and without the cells being deliberately sensitized. This non-adaptive, non-major histocompatibility complex (MHC)-restricted cytotoxicity was defined as ‘natural’ cytotoxicity (Trinchieri, 1989). Later, a system of classification of human cytotoxic lymphocytes was proposed by Lanier and Phillips based on CD3 expression, and whether or not cytotoxicity was MHC restricted (Lanier and Phillips, 1986), leading to CD8^+^ T cells (CTLs) and natural killer (NK) cells, with NK cells further divided into CD3 positive (NKT) and negative populations. A smaller population of ‘unconventional’ T cells defined by expression of the gamma and delta (γδ) T cell receptor chains are also capable of cytotoxic functions (Chien et al., 2014).

Natural killer cells develop from a lymphoid precursor common to B and T cells, but unlike their adaptive cousins, NK cells lack the machinery for somatic recombination and thus do not generate antigen-specific receptors. NK cells instead possess three main sets of receptors that control target cell recognition: natural killer group 2 (NKG2); killer-cell immunoglobulin-like receptors (KIR) and natural cytotoxicity receptors (NCR). These receptor families inhibit or activate NK cell function through a host of target cell surface ligands (Guia et al., 2018). Target cells can undergo loss of MHC-I ligands (known as ‘missing-self’), which normally are inhibitory to NK cells, or alternately NK cells can recognize stress-induced ligands expressed by the target cell (‘self-recognition’). The net result of these interactions is to allow NK cells to define and subsequently remove transformed or damaged cells (Chiossone et al., 2018).

**FIGURE 1:**
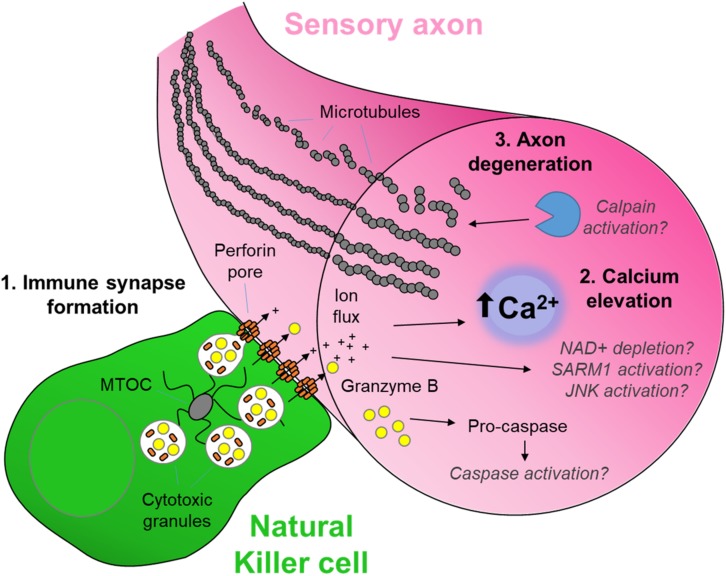
The cytotoxic neuro-immune synapse: Potential downstream intracellular pathways. Cytotoxic natural killer cells form an immunological synapse upon recognition and adhesion to a target, in this case the axon of a stressed sensory neuron. **(1)** Cytotoxic/lytic granules are held by the microtubule organizing center (MTOC) of the NK cell. Immune synapse formation triggers reorientation and polarization of the granules by the MTOC which directs the fusion of the granules to the synaptic membrane. This mechanism ensures the precision release of the cytotoxic contents directly through the targeted axonal membrane. **(2)** The assembly of perforin pore complexes in the axonal membrane from individual perforin subunits released to the extracellular space allows the flow of ions including Ca^2+^, and larger molecules such as the serine protease granzyme B, into the intracellular environment of the target neuron. **(3)** Ca^2+^ flux leads to rapid axon microtubule destabilization and axon degeneration. The full mechanism of cytotoxicity in the axon is unclear. However, in the case of neuron-autonomous Wallerian degeneration, the late-phase Ca^2+^ elevation and subsequent calpain activation required for microtubule destabilization is gated by a metabolic signaling cascade involving depletion of nicotinamide adenine dinucleotide (NAD^+^), activation of Sterile-alpha and armadillo motif containing protein (SARM) and C-Jun N-terminal kinase (JNK) (Llobet Rosell and Neukomm, 2019). Although caspase 3 is a major target of granzyme B protease activity (Adrain et al., 2005), its role in cytotoxic axon degeneration remains unclear. Pathways shown in gray italics are yet to be determined specifically in cytotoxicity-induced axonal degeneration.

**FIGURE 2:**
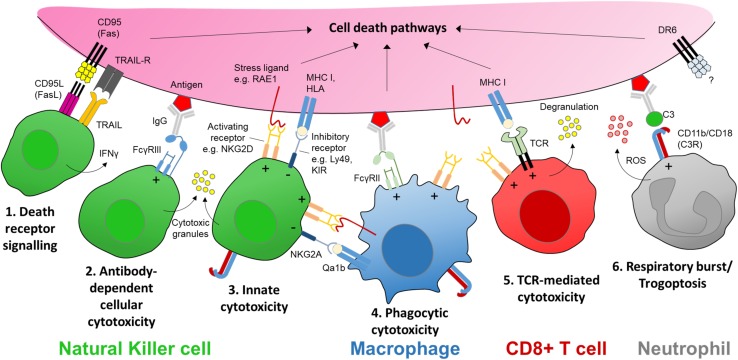
The receptors and ligands of cellular cytotoxicity. There are several key cellular effector mechanisms capable of evoking intracellular cell death pathways. In the case of nerve injury this could be a targeted sensory axon. Natural killer (NK) cells are specialist cytotoxic effector cells expressing multiple receptors and ligands for executing cellular cytotoxicity. **(1)** CD95 (Fas) is a transmembrane protein that belongs to the tumor necrosis factor (TNF) family. Engagement with its ligand CD95L (FasL) expressed on NK cells (as well as activated CD8^+^ T cells and plasma cells) triggers apoptotic signaling in the target cell. Apoptosis may also be induced by tumor necrosis factor-related apoptosis-inducing ligand (TRAIL) via its receptor on target axons. **(2)** Cross-linking of an antigen-antibody complex with the low affinity Fc receptor FcγRIIIa (CD16a) on human NK cells (FcγRIII/CD16 in mice) can trigger cytotoxic granule release (Rosales, 2017). **(3)** NK cells express a host of activating receptors such as NKG2D which recognize ligands expressed at times of neural stress, tumorigenesis or infection. Cytotoxic activity by either Fc or activator receptor function is kept in balance by inhibitory signaling from major histocompatibility complex (MHC) class I molecules or human leukocyte antigens (HLA), present on nearly all somatic cells; these molecules signal through inhibitory receptors expressed on NK cells, e.g., Ly49 family in mice, and killer immunoglobulin-like receptors (KIR) in humans. **(4)** Macrophages may elicit a form of antibody-dependent cellular phagocytosis (ADCP) via engagement with another low affinity Fc receptor FcγRIIa (CD32a) in humans. Macrophages/monocytes may also express natural killer receptors and ligands allowing them to recognize stressed target cells as well as interact with NK cells via natural killer receptor ligands (e.g., RAE1) while protecting themselves from killing by inhibitory MHC I molecules such as Qa1b (in mice) (Zhou et al., 2012). **(5)** Sensitized CD8^+^ T cells recognize MHC I-presented antigens at the target membrane surface via corresponding T cell receptors (TCR), which together with co-stimulation via receptors such as NKG2D, triggers cytotoxic granule release in a manner similar to NK cells. CD8^+^ T cells may also trigger apoptosis in target cells via FasL. **(6)** Neutrophils can recognize opsonized targets, such as antibody-antigen complexes, via complement receptors (e.g., C3R) or Fc receptors including FcγRIIa (Rosales, 2017). Complement or antibody Fc receptor signaling neutrophils can lead to respiratory burst and the release of reactive oxygen species (ROS), or an active mechanical disruption of the target cell membrane known as ‘trogoptosis’ (Matlung et al., 2018). Signaling via death receptor 6 (DR6) is thought to be involved in axon degeneration in the peripheral nervous system as well as neurodegeneration in the brain but the extrinsic signal for the receptor remains unknown (Gamage et al., 2017). ‘**+**’ and ‘**−**’ within the immune cell denotes stimulatory and inhibitory signaling, respectively.

Critical to NK cell cytotoxicity is the recognition and adhesion to the target cell, forming an ‘immune synapse’ (Davis et al., 1999). Target cell engagement is followed by exocytosis and fusion of lytic granules containing pore-forming perforin proteins and serine proteases such as the granzyme family (Henkart, 1985; Figure 1). NK cells can also induce target cell death signaling via activation of intracellular caspase cascades including tumor necrosis factor (TNF)-related apoptosis-inducing ligand (TRAIL) and Fas (CD95/Apo 1)-mediated apoptosis (Zamai et al., 1998). In addition, NK cells can effect target cell removal through antibody-dependent cellular cytotoxicity (ADCC) (Wang et al., 2015; Figure 2).

Human NK cells can be broadly separated into cytotoxic CD56^dim^CD16^+^ cells and cytokine producing CD56^bright^CD16^–^ (Poli et al., 2009); the equivalent populations in mice being CD27^–^CD11b^+^ and CD27^+^CD11b^–^, respectively (Crinier et al., 2018). In addition to cytotoxicity, CD56^bright^ NK cells can shape the immune response in their vicinity by secreting a host of signaling molecules including interferon-gamma (IFN-γ), tumor necrosis factor-alpha (TNFα) and colony-stimulating factor 2 (CSF2) (Vivier et al., 2008; Poli et al., 2009) allowing functional interactions with T cells, dendritic cells and macrophages (Michel et al., 2012; Ferlazzo and Moretta, 2014; Pallmer and Oxenius, 2016). Unlike other innate immune cells, NK cells are not generally considered phagocytic, although phagocytosis of fungal pathogens by NK cells has been reported (Voigt et al., 2014).

Two major families of stress-induced ligands for the activatory receptor NKG2 member D (NKG2D) receptor have been identified in humans: MHC class I chain-related protein A (MICA) and B (MICB), and UL16-binding proteins (ULBP1-6), encoded by the family of retinoic acid early transcript 1 (*RAET1*) genes (Spear et al., 2013). The homologous ligands in the mouse are separated into three families: Histocompatibility 60 (H-60) (Diefenbach et al., 2001), murine UL16-binding protein-like transcript 1 (MULT1) (Carayannopoulos et al., 2002) and the RAE1 proteins family encoded by five *Raet1* genes (α, β, γ, δ and ε) (Cerwenka et al., 2000). NKG2D ligands are often expressed by tumors or virally infected cells (Guia et al., 2018); for example, influenza infection has been shown to upregulate *Raet1* gene expression in mouse sensory neurons *in vitro* (Backstrom et al., 2007). NKG2D ligands may also be expressed by other cell stressors such as during DNA damage or tissue injury (Raulet et al., 2013). The *Raet1* gene family (not to be confused with ribonucleic acid export 1, *Rae1*) was originally identified by its expression in a mouse carcinoma cell line in response to retinoic acid treatment (Nomura et al., 1994).

## NK Cells in the Injured Peripheral Nerve

One of the first descriptions of NK cell-mediated neurotoxicity was in sympathetic ganglion neurons following systemic treatment with guanethidine (Hickey et al., 1992). NK cells harvested from adult mice and stimulated *ex vivo* with the cytokine interleukin-2 (IL-2) were also cytotoxic to dissociated embryonic dorsal root ganglion (DRG) neurons (Backstrom et al., 2000). A clue to the molecular interactions involved was a reduction in DRG cell cytotoxicity by blockade of the NKG2D receptor on NK cells (Backstrom et al., 2003), as well as the high basal expression of *Raet1* in the embryonic sensory neurons (Nomura et al., 1996), which is likely the result of downstream signaling from retinoic acid. Retinoic acid signaling is critical in neurodevelopment (Maden, 2007), providing neurotrophic effects on axonal outgrowth (Corcoran et al., 2000) and acting as a regeneration mediator after nerve injury in adult neurons (Puttagunta and Di Giovanni, 2011).

In contrast to embryonic neurons, *Raet1* expression is minimal in uninjured adult sensory neurons (Backstrom et al., 2000; Davies et al., 2019). Transcripts for *Raet1a, Raet1b* and *Raet1*e, as well as *Ulbp1* (encoding MULT1) and *H60b*, were identified in single DRG neurons isolated from 6 to 8 week old mice, although abundance was low (Usoskin et al., 2015). *Raet1* transcripts are however significantly upregulated in DRG neurons after peripheral nerve injury as detected by whole tissue quantitative-PCR and *in situ* hybridization (Davies et al., 2019). The *Raet1e* transcript specifically was also identified by RNA sequencing of mouse DRG, though it did not reach significance as a differentially expressed gene, likely due to the low abundance at the early time points assessed after injury (<24 h) (Rozenbaum et al., 2018). Additionally, deep sequencing of the rat sciatic nerve showed significant upregulation of *Raet1* 4 days after crush injury (Yi et al., 2015), suggesting either local expression within the injured axon, or additional expression by resident cells within the nerve. Recruitment of NK cells into the injured peripheral nerve (Cui et al., 2000; Hu et al., 2007; Davies et al., 2019) allows for the targeting of RAE1–expressing injured axons for degeneration (Davies et al., 2019) as well as possibly targeting other cell types within the nerve (Yi et al., 2015).

The signaling process driving *Raet1* expression in injured sensory neurons is currently unclear. RAE1 expression during herpes virus infection occurs via the inhibition of histone deacetylase 3 (HDAC3), which normally acts as constitutive repressor of NKG2D-ligand gene expression (Greene et al., 2016). HDAC3 is also exported from the nucleus of injured DRG neurons (Cho et al., 2013) contributing to the histone acetylation which is thought to be necessary for ‘regeneration associated gene’ expression (Cho and Cavalli, 2014). The potential for autoimmune neurodegeneration by NK cells raises the interesting question of epigenetic influences on NKG2D ligand expression as a possible cause of sensory autoimmune neuropathies (Schleinitz et al., 2010). This has been demonstrated in principle by conditional overexpression of *Raet1e* within a population of TRPV1 receptor-positive sensory neurons, which resulted in a loss of heat sensitivity compared to littermate controls, consistent with the absence of peripheral signaling from this important subset of heat-sensitive nociceptive fibers (Davies et al., 2019). *Trpv1* expression in the cell bodies of these sensory nerves was preserved, however, suggesting that the effect of *Raet1e* overexpression occurred in the peripheral axons, much like after injury (Davies et al., 2019). Further work is required to examine the dynamics of the expression of immune ligands within sensory neurons in health and disease.

## NK Cells in Chemically Induced Neuropathies

Peripheral neuropathy is a common side-effect of many chemotherapeutic agents. Axon degeneration occurs after treatment with oxaliplatin or vincristine, despite differences in the mode of action of these drugs (Starobova and Vetter, 2017). Oxaliplatin is a platinum-based chemotherapeutic, which blocks tumor cell replication through DNA binding thus leading to apoptosis, but is also associated with significant toxic side-effects including neurotoxicity (Oun et al., 2018). Accumulation of oxaliplatin in the DRG specifically is thought to contribute to the symptoms of peripheral sensory neuropathy (Sprowl et al., 2013) with functional changes to nerve excitability early in treatment correlating with the severity of neuropathy over time (Park et al., 2009). Vincristine is a vinca alkaloid drug which binds tubulin, resulting in microtubule destabilization and interruption of mitosis in dividing cells (Bates and Eastman, 2017). In sensory neurons, microtubule arrangement alterations are observed particularly in the large myelinated axons of rat sciatic nerves following vincristine treatment but prior to significant axon degeneration (Topp et al., 2000). The mechanisms of peripheral neuropathy by vincristine treatment *in vivo* are likely to involve mitochondrial toxicity and disruption of axonal transport (Fukuda et al., 2017). Neuronal microtubules are highly dynamic and participate in the transport of newly synthesized RNA, proteins and organelles required for nerve homeostasis (Prior et al., 2017). Disruption of axonal transport will therefore likely affect the local expression of RNA and protein that is critical for the efficient wiring and function of developing and regenerating axons (Poulopoulos et al., 2019) and likely explains why the longest axons tend to be affected, resulting in a ‘stocking and glove’ distribution of symptoms at the extremities (Starobova and Vetter, 2017). Non-neuron-autonomous effects from other cell types including immune cells may also contribute to the neuropathic effect of vincristine (Lees et al., 2017).

Chemotherapeutic agents upregulate stress-related proteins in tumor (as well as other) cells, including ligands for NK cells (Zingoni et al., 2017). For example, low-dose chemotherapeutic agents, including oxaliplatin, are capable of upregulating ligands for the activating receptors NKG2D and DNAX accessory molecule-1 (DNAM-1; CD226) and TRAIL in multiple tumor lines leading to enhanced susceptibility to NK cell cytotoxicity (Soriani et al., 2009; Siew et al., 2015). Vincristine and oxaliplatin themselves only mildly impair NK cell cytotoxicity (Markasz et al., 2007). Chemotherapeutic agents can also enhance NK cell function by down-regulation of inhibitory ‘self’ ligands on the target cell surface (Fine et al., 2010); therefore the sensitivity of tumor or other target cells to NK cell cytotoxicity can be initiated by modulation of activating and inhibitory ligands (Quirk and Ganapathy-Kanniappan, 2017). Pre-treatment with the cytokine interleukin 2 (IL-2) increases the anti-tumor response rate to oxaliplatin particularly in patients with low blood lymphocyte counts (Lissoni et al., 2005) but there are currently no data available on how this immune modulation may impact on the neuropathic side effects of chemotherapy.

### Neurotoxicity of Immunotherapies

Immunotherapies that recruit cytotoxic effectors cells (Cifaldi et al., 2017), such as tumor-targeted monoclonal antibodies (mAbs) and checkpoint inhibitors, are also prone to inducing peripheral neuropathic side-effects (Federico et al., 2017; Spain et al., 2017) and in rare cases the inflammatory polyneuropathy Guillain-Barré syndrome (GBS) (McNeill et al., 2019). Tumor targeting by mAbs is fast becoming a mainstay in immunotherapy. Both NK cells and macrophages contribute to ADCC. Binding of the Fc portion of immunoglobulins (IgG) to Fc receptors FcγRIIIa (CD16) or FcγRIIa (CD32) transmits a stimulatory signal to NK cells triggering cytotoxic attack, as well as the release of cytokines such as IFNγ (Cheung and Dyer, 2013; Figure 2). However, the increased use of mAbs has brought an appreciation of significant side effects, in particular autoimmune disease (Hansel et al., 2010) and neurological complications (Bosch et al., 2011). For example, dinutuximab (ch14.18) is a mAb licensed for the treatment for high-risk childhood neuroblastoma and targets the disialosyl glycolipid GD2. NK cells are thought to be the main driver of the anti-tumor effect of neuroblastoma immunotherapy (Main et al., 1985; Lode et al., 1998; Wang et al., 2015) such that gain of NK cell function is often sought in combination with antibody treatment (Koehn et al., 2012). The combination of anti-GD2 mAbs with IL-2 improves outcomes in high-risk neuroblastoma patients suggesting that cellular effectors, in particular NK cells, are key to this response (Yu et al., 2010). However, during anti-GD2 mAb immunotherapy patients experience significant neuropathic pain (Cheung et al., 1987) and in severe cases irreversible peripheral sensory neuropathy. Anti-GD2 mAbs recognize the GD2 antigen on axons and affect peripheral nerve function *in vivo* (Slart et al., 1997; Xiao et al., 1997) but how this occurs remains unclear. The anti-tumor effect of anti-GD2 antibodies involves activation of the complement system (complement-dependent cytotoxicity, CDC) in addition to ADCC (Imai et al., 2005). Research has until now focused on antibody modifications designed to reduce complement activation, which has had some success in reducing neuropathic side effects in rat models (Sorkin et al., 2010). However, trials with newer mAb isoforms with impaired complement activity still report significant peripheral nerve toxicities (Navid et al., 2014; Anghelescu et al., 2015; Federico et al., 2017), suggesting that other mechanisms of mAb-induced neuropathy are at play (Calvo et al., 2012). Although immune-mediated inflammation has been proposed as a possible cause of chemotherapy and immunotherapy-induced pain (Starobova and Vetter, 2017), the role of cellular cytotoxicity in these pathologies requires further investigation.

## Axon Degeneration and Cell Death Pathways in Nerve Injury

Wallerian degeneration, the process responsible for axonal degeneration following nerve axotomy *in vivo* and in the severed axons of cultured sensory neurons *in vitro*, is an active processes intrinsic to the neuron (Lunn et al., 1989; Perry et al., 1990). The discovery of a spontaneous mouse mutation with delayed or ‘slow’ Wallerian degeneration (*WldS*) demonstrated that over-activity of the nicotinamide adenine dinucleotide (NAD^+^) biosynthetic enzyme NMNAT1, is sufficient to protect the severed axon from fragmentation (Araki et al., 2004; Sasaki et al., 2009). A *Drosophila* screen of axon degeneration after olfactory neuron axotomy led to the identification of dSarm and its mammalian ortholog: Sterile alpha and TIR motif-containing 1 (SARM1) (Osterloh et al., 2012). SARM1 rapidly breaks down NAD^+^ and therefore acts as the key regulator of axonal degeneration after injury (Gerdts et al., 2015; Figure 1). SARM1 has been proposed to be a point of convergence for axonal degeneration associated with chemotherapy (Geisler et al., 2016) and metabolic neuropathies (Turkiew et al., 2017) but interestingly, not neuronal death (Geisler et al., 2019a).

**FIGURE 3:**
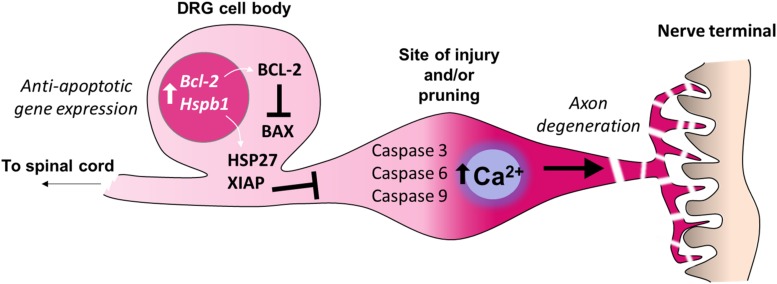
Inhibition of apoptosis in the cell bodies of sensory neurons. Adult sensory neurons are protected from cell death by expression of *Bcl2* (B-cell lymphoma 2). The gene product BCL-2 suppresses the function of the pro-apoptotic protein BAX (BCL2 Associated X) which can be induced by cell death signaling. The expression of further anti-apoptotic genes in the cell bodies of sensory neurons prevent axon degeneration intracellular signaling cascades from causing neuronal death. For example, expression of *Hspb1* (Heat shock protein family B1), also known as HSP27, prevents sensory neuron death after nerve injury. X-linked inhibitor of apoptosis protein (XIAP) prevents cell death during developmental axon pruning by inhibiting caspases in the cell body. Thus axonal activation of caspases 3, 6 and 9, which is sufficient for certain forms of axon-restricted degeneration, occurs in the absence of cell apoptosis (Simon et al., 2012; Cusack et al., 2013).

Axonal loss may also occur in intact neurons by pruning or dying back. These processes share many features with Wallerian degeneration such as disruption of microtubules and cytoskeleton rearrangement (Luo and O’Leary, 2005). However, in contrast to the Wallerian degeneration of severed axons, axonal pruning during development uses the machinery of apoptosis while avoiding neuronal death via the endogenous caspase inhibitor, X-linked inhibitor of apoptosis protein (XIAP) (Unsain et al., 2013; Yaron and Schuldiner, 2016; Figure 3). The degree of peripheral axon pruning after injury may also be driven by a combination of trophic and axon guidance factors, which also co-opt apoptotic pathways (Vanderhaeghen and Cheng, 2010).

Genes related to programed cell death have long been known to be regulated within the dorsal root ganglia (DRG) of injured sensory neurons (Costigan et al., 2002). For example, the elevation of pro-apoptotic caspases occurs in injured DRG neurons as well as surrounding non-neuronal glial cells (Vigneswara et al., 2013; Wiberg et al., 2018), and single cell analysis of sensory ganglia shows that caspase 3 is upregulated in all sensory neuron subtypes after injury (Hu et al., 2016). In fact more than 10 cell death-related genes are among the top 438 genes commonly induced by nerve injury in more than 5 sensory neuron subtypes revealed by single cell gene ontology analysis (Renthal et al., 2019). Other cell death-related genes, such as programed cell death 2 (*Pdcd2*) are upregulated only in the non-peptidergic (NP) population of nociceptive neurons; conversely, NP neurons showed a *down*-regulation of the neuronal survival related genes ISL LIM homeobox 1 (*Isl1)* and oxidation resistance 1 (*Oxr1*) (Hu et al., 2016), suggesting a selective sensitivity of this nociceptive neuron subpopulation to cell-death mechanisms.

While substantial neuronal death can occur in the DRG in neonatal animals after nerve injury (Himes and Tessler, 1989), such sensory loss is limited in adults (Whiteside et al., 1998; Tandrup et al., 2000; Shi et al., 2001). Nerve injury in young animals (up to 3 weeks of age) results in a reduced expression of the neuroprotective anti-apoptotic gene *Bcl-2* (Gillardon et al., 1994; Farlie et al., 1995); this developmental period correlates with the lack of neuropathic pain development following peripheral nerve injury (Howard et al., 2005; Costigan et al., 2009a; Fitzgerald and McKelvey, 2016). In adults, DRG expression of *Bcl-2* remains high (Merry et al., 1994) regardless of nerve injury type (Gillardon et al., 1994). The upregulation of heat shock proteins such as HSP27 (encoded by *Hspb1*) also support neuronal survival after axotomy (Costigan et al., 1998; Lewis et al., 1999; Figure 3). Conversely, after peripheral nerve transection, adult mice deficient in the pro-apoptotic gene *Bax* display greater autotomy (self-mutilation) (Lyu et al., 2017), a sign of ongoing neuropathic pain (Bennett and Xie, 1988), suggesting that certain apoptotic or ‘cell death’-related pathways may in fact be pain-protective in the face of nerve injury. Similarly, cross-linking of the death receptor Fas (also known as CD95) on sensory neurons enhances neurite outgrowth and nerve regeneration after sciatic nerve crush (Desbarats et al., 2003). Upregulation of Fas ligand within the injured nerve (Li et al., 2018) is therefore likely to have an important bearing on the resolution of nerve injury via non-apoptotic signaling to neurons (Desbarats et al., 2003) and immune cells (Williams et al., 2018) in addition to apoptotic mechanisms (Li et al., 2018; Figure 2).

Despite the role of SARM1 as the gate-keeper of intrinsic axon degeneration after injury (DiAntonio, 2019), extrinsic factors also influence axon degeneration (Conforti et al., 2014). For example, recent evidence points to the potential of extracellular signaling driving Wallerian degeneration through the orphan death receptor 6 (DR6) (Gamage et al., 2017). The cytotoxic machinery of NK cells is also well-placed to initiate axon degeneration. Immune-synapse formation with a target cell, followed by perforin pore formation and granzyme entry to the cytoplasm, typically triggers a cascade of calcium-dependent signaling events which in many cell types then leads to apoptosis (Trapani and Smyth, 2002). In sensory neurons, increases in axonal calcium either through the perforin pore or released from intracellular stores, appears critical to the subsequent localized axon degeneration by NK cells (Davies et al., 2019; Figure 1). The ability of calpain inhibitors to prevent axonal degeneration after nerve injury (McGonigal et al., 2010; Ma et al., 2013), as well as injury by complement-mediated membrane attack complex (MAC) pore formation (O’Hanlon et al., 2003), suggests that calcium-dependent processes are sufficient for degeneration. Thus the formation of a ‘neuro-immune synapse’ and its consequent actions within the axon represents an important extrinsic mechanism of axon degeneration in the periphery (Figure 1). The exact mechanisms downstream from granzyme entry into the axoplasm and how these interact with the known intrinsic pathways of axon degeneration, including SARM1, are currently unknown.

### Axon Degeneration and Neuropathic Pain

The relationship between axon degeneration and neuropathic pain is complex. Experiments in Wallerian degeneration null mice show that while chronic constriction injury and chemotherapy-induced ‘pain’ does appear to require axon degeneration (Ramer et al., 1997; Geisler et al., 2016), other nerve injury models, such as spinal nerve transection, do not (Ramer and Bisby, 1998). On the other hand, complete nerve crush injury inducing Wallerian degeneration of the injured axons results in less long-term mechanical hypersensitivity than a partial or ‘moderate’ crush injury (Huang et al., 2012; Davies et al., 2019). Thus it appears that axon degeneration is neither necessary nor sufficient for neuropathic pain. Instead, the relative proportion of the injured and uninjured axon fibers and their functional subtypes or modalities is a more likely determining factor, reflective of the fact that most pre-clinical neuropathic pain models are based on a partial injury of the nerve (Seltzer et al., 1990; Kim and Chung, 1992; Decosterd and Woolf, 2000). In addition, neuropathies as a result of physical trauma, metabolic or chemical injury result in an intermediate state of injury between normal and axotomized, as evidenced by the pathological changes observed in axons the absence of complete Wallerian degeneration (Topp et al., 2000; Park et al., 2009; Huang et al., 2012; Davies et al., 2019). Axons in such a ‘metastable’ state of injury (Williams et al., 2014), may also be defined by the expression of cell-stress markers, including immune ligands, on their membranes (Davies et al., 2019). These findings suggest that peripheral nerve recovery may be achieved by either axonal stabilization (Geisler et al., 2019b) if intervention is performed early, or by accelerated degeneration if significant injury has already occurred (Davies et al., 2019).

## Granzymes in Neuropathy

Granzymes are a family of serine proteases produced by cytotoxic NK and T cells that have recently been linked to nerve injury and pain. Granzyme B is detected within the mouse peripheral nerve after transection and crush injuries, of which NK cells are a significant source (Davies et al., 2019). *In vitro*, the presence of granzyme B in the culture media is not sufficient to induce DRG axon toxicity, indicating that granzymes are delivered to the axon cytosol by direct contact between cytotoxic cells and sensory nerves (Davies et al., 2019). This suggests the formation of a neuro-immune synapse is critical in the neurodegenerative effect of cellular cytotoxicity by NK cells (Figure 1). In clinical neuropathies, granzymes have been labeled in the peripheral nerves (Noguchi et al., 2005; Ni Mhaolcatha et al., 2019), and granzyme B positive lymphocytes are seen in close apposition to neurons in the DRG in the late-stages of Guillain-Barré syndrome (GBS) in which axon degeneration is observed (Wanschitz et al., 2003). In the central nervous system (CNS) granzyme B-positive cells can be found next to apoptotic neurons following stroke (Chaitanya et al., 2011). Further studies are required to determine the exact role of these cytotoxic effector proteins following nerve injury.

Besides direct cytotoxicity, another potential target for granzymes are protease-activated receptors (PARs), a number of which have been implicated in pain pathogenesis (McDougall and Muley, 2015). For example, PAR-4 activation by the serine protease cathepsin G is capable of suppressing sensory neuron excitability (Sessenwein et al., 2017). Granzymes have been implicated in pain phenotypes more broadly, for example in a genome wide association study (GWAS) of healthy human twins the gene *GZMM*, encoding granzyme M, was identified as strongly associated to thermal nociception (Williams et al., 2012). Conversely, an inhibitor of granzyme B, serpinA3N, is produced by DRG neurons after peripheral nerve injury (Vega-Avelaira et al., 2009) where it is thought to attenuate mechanical allodynia in mice by inhibition of another serine protease, leukocyte elastase (Vicuna et al., 2015). Treatment of mice with small molecule inhibitors of leukocyte elastase after nerve injury temporarily reduced established neuropathic mechanical allodynia (Bali and Kuner, 2017). Furthermore, serpinA3N is capable of rescuing human fetal neuronal cell death mediated by anti-CD3 and anti-CD28 activated T cells (Haile et al., 2015). What role serpinA3N or other serine protease inhibitors may have in mitigating cytotoxic effector functions in the peripheral nerve after injury remains to be explored.

## Cellular Conspirators in Axonal Degeneration

### Macrophage Cytotoxicity in Nerve Injury

Natural killer cells now join macrophages as immune cells participating in the degeneration process after peripheral nerve injury (Chen et al., 2015). Macrophages are phagocytic cells that may be tissue resident or originate from the monocyte population of the blood and are an important component of the innate immune response to nerve injury (Zigmond and Echevarria, 2019). Macrophages play a key role in the removal and degradation of myelin debris during Wallerian degeneration (Stoll et al., 1989), and appear capable of degenerating both myelinated and unmyelinated fibers, as liposome depletion of macrophages prior to partial nerve ligation led to a preservation of both fiber types (Liu et al., 2000). However, macrophages do not appear capable of overriding the intrinsic delay in Wallerian degeneration in the *WldS* mouse despite their recruitment to the injured nerve (Rosenberg et al., 2012). In the absence of frank nerve injury, however, macrophages could play an active role in the peripheral axon degeneration that occurs in aged mice (Yuan et al., 2018).

Monocytes and macrophages exert cytotoxicity via cell-contact dependent phagocytosis (Munn and Cheung, 1990; Gul et al., 2014; Figure 2). Neuron morphology may be affected by the phagocytosis of viable axons and dendrites, in a process termed ‘neurophagy’ (Vilalta and Brown, 2018). After peripheral nerve injury for example, both central and peripheral projections of primary afferent neurons display signals for phagocytosis by microglia (Maeda et al., 2010) and macrophages (Wakatsuki et al., 2017), respectively. Macrophages can recognize the loss of sialic acid residues on membrane-bound glycolipids (desialylation) leading to the removal of neurites by phagocytosis (Linnartz-Gerlach et al., 2016). Neurite phagocytosis by macrophages requires the cell adhesion molecule CD11b, part of the complement C3 receptor (CR3), which recognizes deposition of the complement component C1 on desialyated glycolipids (Linnartz-Gerlach et al., 2016). Thus the complement system works in synergy with innate immunity to mark neuronal membranes for removal. In a model of simian immunodeficiency virus (SIV) infection, monocyte chemotaxis within the DRG led to neuronal loss which correlated the loss of intraepidermal nerve fibers (IENF) (Lakritz et al., 2015). Conversely, sialylation of DRG neuronal cell bodies after injury may help to prevent cytotoxic attack (Peng et al., 2004) by engaging inhibitory signaling via SIGLECs (sialic acid-binding immunoglobulin-type lectins) on innate immune cells, including NK cells and monocytes (Nicoll et al., 1999; Crocker and Varki, 2001).

### Neutrophil Cytotoxicity in Nerve Injury

Neutrophils respond to tissue injury within minutes by sensing necrotic cell debris and damage-associated molecular patterns (DAMPs) (Wang, 2018). Initial contact by patrolling neutrophils results in their death by apoptosis and recruitment of a neutrophil swarm (Kienle and Lammermann, 2016). Neutrophil swarming often leads to collateral damage, although cloaking of the damage site by tissue resident macrophage may help to limit the inflammatory cascade (Kienle and Lammermann, 2016; Uderhardt et al., 2019). Neutrophil accumulation requires IL-1 and TNF receptor signaling, probably via IL-1β and TNFα released from resident cells early after injury (Nadeau et al., 2011). Neutrophil depletion experiments confirm their importance in the subsequent development of hyperalgesia after nerve injury (Perkins and Tracey, 2000; Nadeau et al., 2011). Blockade of CXCL1 cytokine signaling led to a reduction in neutrophil recruitment, and also reduced mechanical hyperalgesia in a model of post-surgical pain (Carreira et al., 2013) although the source of CXCL1 in the nerve and DRG after injury remains unclear (Silva et al., 2017).

Although traditionally known for amplifying the inflammatory response to tissue injury, neutrophils also contribute to tissue repair by the release of growth factors, and polarizing phagocytic macrophages to release anti-inflammatory cytokines such as TGFβ and IL-10 (Wang, 2018). Neutrophils appear to be critical for regeneration of CNS axons, such as after optic nerve crush (Kurimoto et al., 2013), although in the peripheral nervous system no significant deficits in recovery from sciatic nerve injury were observed after neutrophil depletion (Nadeau et al., 2011). More recent evidence suggests that in the absence of infiltrating monocyte-derived macrophages, neutrophils also perform a significant phagocytic role in the injured peripheral nerve (Lindborg et al., 2017).

Neutrophils can release reactive oxygen species (ROS) in a metabolic event known as a respiratory burst, or release of neutrophil extracellular traps (NETs) in a cell death processes known as NETosis. These cytotoxic mechanisms are typically targeted at invading pathogens but may be dysregulated in immune-related disease (Papayannopoulos, 2018). A novel form of cytotoxicity enacted by neutrophils has recently been described. Clearance of antibody-coated or ‘opsonized’ cancer cells, termed ‘trogoptosis,’ results from target cell membrane disruption leading to necrotic death (Matlung et al., 2018). Opsonization of peripheral nerve components such as myelin may occur after injury either by coating with IgG (Vargas et al., 2010) or complement C3 (Bruck and Friede, 1991). The C3 receptor (also known as CD11b/CD18) is expressed by NK cells, macrophages, monocytes and granulocytes (Arnaout, 1990) as well as neutrophils, where it has been shown to be critical for immune synapse formation and ADCC (van Spriel et al., 2001; Figure 2). Thus the same processes known to mediate phagocytosis of cellular debris in the injured nerve may serve the dual purpose of engaging cell death pathways by infiltrating immune cells.

### Schwann Cell Cytotoxicity in Nerve Injury

Schwann cells, which form the myelinating units of the peripheral nerve, respond dynamically within minutes of injury (Wong et al., 2017). Schwann cells collaborate with macrophages to phagocytose degenerated myelin after nerve injury utilizing both Tyro3-Axl-Mer (TAM) receptor-mediated and autophagic mechanisms (Brosius Lutz et al., 2017). Recent evidence suggests that Schwann cells may themselves be early participants in peripheral axon degeneration (Catenaccio et al., 2017), and delaying dedifferentiation of Schwann cells prolongs the survival of axons after crush injury (Catenaccio et al., 2017). The presence of newly discovered Schwann cells adjacent to free nerve endings in the skin are ideally placed to mediate in the dying back of nerve terminals that occurs in multiple peripheral neuropathies (Abdo et al., 2019). However, the role of Schwann cells in triggering Wallerian degeneration remains unclear.

### T Cell Cytotoxicity in Nerve Injury

Peripheral nerve injury recruits CD8^+^ T cells to the injured sciatic nerve (Hu et al., 2007; Bali and Kuner, 2017) where expression of antigen-presenting MHC class I molecules are increased (Bombeiro et al., 2016). Although sensory neurons express IFN-γ receptors (typically required for MHC I induction) they fail to upregulate MHC class I in response to IFN-γ signaling (Turnley et al., 2002). Correspondingly, MHC class I expression in axons of the injured nerve is low (Bombeiro et al., 2016). Therefore, the mechanism of CD8^+^ T cell activation by MHC class I induction and antigen presentation after acute nerve injury is unclear. *In vitro*, antigen-specific CD8^+^ T cells are known to cause axonal injury (Meuth et al., 2009; Sauer et al., 2013). Lentivirus infection of sensory neurons leads to damage by CD8^+^ T cells via CD40-CD154 mediated signaling (Zhu et al., 2006). Conversely, trigeminal ganglion neurons infected with herpes simplex virus type I are resistant to apoptosis induced by CD8^+^ T cells (Knickelbein et al., 2008), suggesting that the mechanisms controlling T cell mediated neurodegeneration are context dependent.

CD8^+^ T cells (as well as γδ T cells) express the receptor NKG2D and engagement with ligands such as RAE1 augments CD8^+^ T cell function, but only after prior stimulation by antigen-specific T cell receptors (TCRs) (Lanier, 2015), suggesting that NK cells are an important responder to ‘stressed-self’ in the non-immunized state. NK and CD8^+^ T cells exhibit mutually antagonistic responses to IL-2 immune stimulation (Alvarez et al., 2014) meaning that despite their overlapping cytotoxic roles one or other population will tend to dominate in any given immune response. An example of this is the aberrant expression of RAE1 in pulmonary epithelial cells, which has previously been shown to contribute to chronic obstructive pulmonary disease (COPD) via cytotoxic lymphocytes in an NK but not T-cell dependent manner (Borchers et al., 2009). Similarly, in the case of peripheral nerve injury NK cells are sufficient for degeneration of axons after IL-2/anti-IL-2 antibody complex stimulation (Davies et al., 2019) despite the simultaneous expansion of CD8^+^ T cells (Boyman et al., 2006). However, additional roles for CD8^+^ T cells within the nerve post-injury cannot be ruled out. Interestingly, resolution of neuropathic pain in mouse models of chemotherapy-induced neuropathy requires CD8^+^ T cells (Krukowski et al., 2016; Laumet et al., 2019a); in these studies the authors propose an indirect involvement of CD8^+^ T cells in suppressing DRG neuron activity by upregulation of IL-10 signaling (Krukowski et al., 2016). T cells are frequently identified in cellular and genetic analysis of peripheral neuropathies (Moalem et al., 2004; Costigan et al., 2009a; Vicuna et al., 2015; Cobos et al., 2018) and there is mounting evidence of their involvement in numerous pain states (Laumet et al., 2019b). Whether such an association between pain and T-cells require cytotoxic effector functions remains to be determined.

## Evidence of NK Cell Function in Pain Syndromes

Acute pain (self-administered painful electrical stimulation) transiently increases functional NK cell activity as well as the proportion of NK cells in peripheral blood (Greisen et al., 1999); this effect was abolished by application of a local anesthetic to the skin, suggesting a neurogenic activation of NK cells (Greisen et al., 1999). NK cell function can also be stimulated by electro-acupuncture in rats (Kim et al., 2005). Peripheral nerve injury increased blood NK cytotoxicity in the 1st week after injury in mice (Kang et al., 2007), whereas splenic NK function assessed 3 weeks after nerve ligation was reduced (Sunagawa et al., 2000) suggesting a window of time after injury in which NK function is active followed by either recovery or exhaustion.

Gene ontology analysis of differentially expressed genes (DEG) shows significant upregulation of NK cell cytotoxicity-related genes after peripheral nerve injury (Costigan et al., 2009a) and during myelination *in vitro* (Wu, 2018). Cell senescence in the aging vertebral disc, a risk factor for low back pain, has also recently been associated with a differential expression of NK cell mediated cytotoxicity-related genes (Liu et al., 2018). Few studies have directly analyzed NK cells in pain conditions in humans. Low back pain patients presented with a lower percentage of NK cells in peripheral blood than asymptomatic subjects (Brennan et al., 1994), while recent studies in a similar cohort of chronic lower back pain patients identified an increase in anti-inflammatory T regulatory (T_reg_) cells (Luchting et al., 2014, 2015). In the context of tumor immunotherapy, the efficiency of NK cell cytotoxicity benefits from a simultaneous suppression of T_reg_ cell function (Ni et al., 2012; Littwitz-Salomon et al., 2015). Similarly an imbalance in NK and T_reg_ cells leading to loss of NK cell function by the release of immuno-suppressive cytokine such as TGF-β may contribute to low back pain (Luchting et al., 2014, 2015). On the other hand, treatment of radicular pain patients with pulsed radiofrequency to the DRG, which led to improved pain outcomes, reduced NK cell frequency in cerebrospinal fluid (CSF) (Das et al., 2018), although peripheral blood counts were not reported.

### Immune Phenotyping After Injury or Pain

A number of studies have used mass cytometry to assess immune cell subsets, including NK cells, in patients after surgery or trauma. In patients undergoing hip arthroplasty for non-traumatic osteoarthritis NK cells increased 1.6-fold in the blood within an hour of surgical trauma, followed by contraction at 24 and 72 h (Gaudilliere et al., 2014). In a study of severe trauma patients, NK cells expressing the transcription factor T-bet peaked in the blood 1 day after injury suggesting the recruitment of immature NK cells from the bone marrow (Seshadri et al., 2017). Traumatic brain injury resulted in a higher frequency of inhibitory receptor (KIR2D^+^NKG2A^+^) expressing NK cells in the circulation compared to healthy or major surgery controls, which interestingly correlated with a loss of HLA class I in monocytes and impaired cytotoxicity to missing-self (Roquilly et al., 2017). While these studies suggest a significant alterations of NK cell function in the aftermath of injury, including to the central nervous system, the NK cell phenotype has yet to be explored in patients with defined injury to a peripheral nerve.

Recent studies with more broadly defined chronic pain conditions failed to find an association between NK cell frequencies and pain groups. The percentage of CD56^bright^CD16^+^ cells were slightly increased in patients with broadly defined severe chronic pain, however, generalized NK cell cytotoxic activity and the frequency of the major NK cell subsets were not significantly different (Yoon et al., 2018). In a study of 14 patients with complex regional pain syndrome (CRPS) and 14 controls, Russo and colleagues used mass cytometry to identify an expansion of both CD4^+^ and CD8^+^ memory T lymphocytes in patients compared to controls (Russo et al., 2019). NK cells (defined as CD56^+^ CD19^–^CD3^–^) were more than 50% elevated in CRPS patient blood but this did not reach significance. Thus results from human studies appear to show regulation of NK cells in chronic pain conditions, though the correlation between NK cell function and pain outcomes remains unclear. The inability to assign function from cell numbers alone is likely due to positive and negative effector functions and diverse cell subsets (Eberl et al., 2015). Appreciation of the differential NK cell subsets that are present in the blood, splenic compartments and injured nerves of mice and humans (Crinier et al., 2018) will aid further deep phenotyping to build a clearer picture of NK cell function in chronic pain.

There is no difference observed in the numbers of nerve-infiltrating NK cells in rats with mechanical allodynia after injury compared to those without (Cui et al., 2000). Furthermore, mice lacking NK cells after chronic depletion maintain a robust development of thermal and mechanical allodynia (Davies et al., 2019) in a well characterized model of neuropathic nerve injury (Kim and Chung, 1992). Therefore, NK cells do not appear to be necessary for the development of neuropathic pain. Instead, various factors may affect the pain-resolving potential of NK cells in the period after nerve injury (Davies et al., 2019). For example, long-term over-expression of NKG2D ligands such as *Raet1* is known to cause a down-regulation of NK cell receptor expression and loss of function (Morvan et al., 2017). Additionally, activating ligands may be shed from damaged nerves, preventing their removal by NK cells. For example, cleavage of NKG2D ligands on tumor cells by disintegrins and metalloproteinases (ADAMs) (Waldhauer et al., 2008) helps tumors escape immuno-surveillance by NK cells (Ferrari de Andrade et al., 2018). As well as functions related to extracellular matrix remodeling (Yong, 2005), matrix metalloproteinases (MMPs) may affect the neuro-immune balance through the regulation of immune ligands on neurons and other cells of the nervous system. Specifically, MMP-9 and MMP-14 are capable of the cleavage of NKG2D ligands (Liu et al., 2010; Sun et al., 2011) are upregulated after nerve injury and contribute to neuropathic pain in preclinical models (Kawasaki et al., 2008; Tonello et al., 2019). Certain MMP gene variants have also been associated with the risk of low back pain in patients (Tegeder and Lotsch, 2009; Bjorland et al., 2017).

Natural killer cell function after nerve injury may also be compromised by morphine, which suppresses both spontaneous and cytokine-stimulated NK cell functions (Yeager et al., 1995). Immune suppression in response to chronic pain and neurological stress has long been known to affect tumor immunity and mortality (Liebeskind, 1991). Chronic stress paradigms, including surgical stress, reduces NK cell cytotoxic function in rodents (Aarstad et al., 1983; Pollock et al., 1987; Ben-Eliyahu et al., 1999) in an opioid-dependent manner (Shavit et al., 1984). Immune suppression after traumatic injury in patients, shown to be related to surgical stress (Blazar et al., 1986; Pollock et al., 1991), could have an important bearing on pain outcomes by reducing the anti-neuropathic function of cytotoxic effectors.

## NK Cell-Macrophage Crosstalk in Pain?

Macrophages have a recognized involvement in pain hypersensitivity with multiple subtypes persisting within the nerve many months after injury (Liang et al., 2019). Depletion of peripheral monocytes/macrophages in mice by systemic treatment with clodronate liposomes reduced degeneration of both myelinated and unmyelinated fibers following nerve injury (Liu et al., 2000) and these mice also showed a reduced incidence of pain (Liu et al., 2000; Cobos et al., 2018). Incidence of neuropathic mechanical allodynia was also delayed by macrophage depletion in a rat model of diabetes (Mert et al., 2009). Activation of the angiotensin type 2 receptor (AT2R) in macrophages can drive neuropathic pain (Shepherd et al., 2018b). The cellular functions of macrophages within the injured nerve may include the production of reactive oxygen species (ROS) leading to nociceptor sensation (Shepherd et al., 2018a) as well as inflammatory cytokine production (Arango Duque and Descoteaux, 2014). Inflammatory macrophages (M1) within the nerve are also implicated in pain associated with diabetic neuropathy (Saika et al., 2019). M1 skewed macrophages within and around the damaged nerve likely precipitate neuropathic symptoms (Kiguchi et al., 2017; Saika et al., 2019) and conversely, macrophage polarization to an anti-inflammatory ‘M2’ phenotype can attenuate neuropathy-induced mechanical hypersensitivity via the production of opioids (Kiguchi et al., 2015; Pannell et al., 2016). Patients with complex regional pain syndrome show an elevation of CD14^+^ CD16^+^ monocytes in their blood (Ritz et al., 2011) suggesting that altered macrophage function at the systemic level is functionally related to nociplastic pain, i.e., pain that arises from altered nociceptive function in the absence of obvious precipitating cause (Aydede and Shriver, 2018). Transcriptomic analysis of human DRG from neuropathic pain sufferers suggests that sex differences in pain susceptibility may be driven by differential gene expression in macrophages (North et al., 2019). Interestingly, the transcriptomic profile of macrophages in mice differs between nerve and DRG compartments (Liang et al., 2019), and recent experiments using tissue-targeted macrophage depletion suggest that macrophages in DRG but not the sciatic nerve are required for mechanical allodynia after nerve injury (Yu et al., 2020). The divergence of neuropathic pain development in young and adult animals may also be a function of differential functional interactions between macrophage and cells within the DRG (Vega-Avelaira et al., 2009) or microglia/T cell signaling in the CNS (Sorge et al., 2015).

In mice, activated macrophages express NKG2D (Zhou et al., 2012) suggesting that in addition to NK cells, macrophages may interact with damaged axons via the expression of the ligand RAE1 after traumatic injury (Davies et al., 2019; Figure 2). Macrophages and NK cells communicate through a wide variety of cell contact cues and soluble factors (Michel et al., 2012). Functional influence is two-way, with macrophages able to either activate or inhibit NK cells (Nedvetzki et al., 2007; Krneta et al., 2017), and NK cells capable of killing over-stimulated macrophages (Nedvetzki et al., 2007). Thus activated NK cells in the immune milieu of the injured nerve may additionally help resolve neuropathic pain by eliminating M1 macrophages (Nedvetzki et al., 2007) as well as damaged axons (Davies et al., 2019).

Endometriosis is another example of a disease that may be influenced by a functional interaction between NK cells and macrophages. Endometriosis is the ectopic growth of endometrial cells forming lesions within the peritoneal cavity surrounding the uterus (Symons et al., 2018) leading to chronic pelvic pain and infertility (Thiruchelvam et al., 2015). A higher density of nerve fibers surrounded by macrophages are observed in endometrial lesions relative to surrounding tissue (Tran et al., 2009) and growth factor production by these cells has recently been proposed as a key driver of neurogenesis and nerve fiber sensitization in these lesions (Forster et al., 2019). Although inflammatory cytokines such as TNFα are elevated in the peritoneal cavity in painful endometriosis (Scholl et al., 2009), TNFα antagonists are yet to show clinical efficacy (Lu et al., 2013). Endometriosis pathophysiology is thought to involve a failure of NK cells to eliminate endometrial lesions from the peritoneal cavity (Sikora et al., 2011). Peripheral blood and peritoneal NK cells show reduced cytotoxic function in women with endometriosis (Garzetti et al., 1993; Thiruchelvam et al., 2015), and downregulation of NK cell cytotoxic capability via inhibitory interactions with macrophages (Yang et al., 2017). Disease severity is correlated with a loss of NK cell cytotoxicity as well as a resistance of the endometrial lesions to NK mediated attack (Oosterlynck et al., 1991). A failure of NK cell cytotoxicity to target aberrantly sprouting peripheral nerves in the presence of activated macrophages could therefore contribute to the symptoms of this disease. Early clinical trials of recombinant IL-2 treatment in endometriosis showed lower recurrence of pain symptoms (Acien et al., 2003), suggesting the potential benefit of the anti-neuropathic function NK cells (Davies et al., 2019). A registered trial aimed at assessing the potential use of an inhibitor of programed cell death 1 (PD-1) could lead to an immunotherapeutic treatment for endometriosis^1^.

## NK Cells in Hematological Malignancies and Inflammatory Neuropathies

Interactions between NK cells and peripheral nerves precipitating neuropathy may also occur as a result of transformation of NK cells themselves. A case study in the 1990’s first reported an NK cell-specific leukemia with associated peripheral neuropathy presenting as tingling hands and back pain with progressive sensory loss in the extremities (Bobker and Deloughery, 1993); sural nerve biopsy revealed heavy lymphocytic infiltration although NK cell identity could not be confirmed (Bobker and Deloughery, 1993). Later, NK cell lymphoproliferative disease (NK-LPD) was reported as associated with peripheral neuropathy (Leitenberg et al., 1995), with steroid treatment improving neurological symptoms concurrent with a reduction in NK cell counts (Leitenberg et al., 1995). Prolonged F-wave latencies were consistent with a demyelinating sensorimotor polyneuropathy and sural nerve biopsies revealed an inflammatory neuropathy with myelin loss (Leitenberg et al., 1995). Subsequent cases of peripheral neuropathy coincidental to the NK lymphocytosis have been reported (Lackowski et al., 1998; de Boer et al., 1999; Noguchi et al., 2005). The effectiveness of steroids (Leitenberg et al., 1995; Sano et al., 2017) or an anti-CD52 antibody targeting a broad population of lymphocytes including NK cells (Chee et al., 2009) for the resolution of the peripheral lesions suggests the involvement of cellular immunity in the neurological aspect of the disease (Leitenberg et al., 1995). Nerve biopsies within these patients revealed lymphocytic infiltrates (Bobker and Deloughery, 1993; Rabbani et al., 1999) and labeling of granzyme and perforin suggest these cells possess cytotoxic activity (Noguchi et al., 2005). A recent observation of CD3^–^ CD56^+^ cells in cutaneous nerve infiltration secondary to NK-LPD appears to confirm their NK cell identity (Ni Mhaolcatha et al., 2019). A longitudinal study of a severe polyneuropathy was additionally characterized by elevated serum levels of IL-2 and TNFα, as well as increased intrinsic NK cell cytotoxicity (Wex et al., 2005).

Overall, 3% of chronic NK-LPD cases appear to go on to develop peripheral neuropathy (Poullot et al., 2014). The reason for the development of neuropathy with NK cell proliferation in a minority of patients is currently unknown. NK cell leukemia is thought to be caused by a dysregulation of survival signals, including gain-of-function mutations of the transcription factor STAT3, leading to cell proliferation (Lamy et al., 2017). NK cells from lymphoproliferative disorders possess a skewed pattern of KIR gene expression (Hoffmann et al., 2000; Epling-Burnette et al., 2004) suggesting either a propensity for over-activation or loss of constitutive inhibition. Prior infection with Epstein-Barr virus (EBV) is also implicated in the pathogenesis of an aggressive form of NK-LPD (Hart et al., 1992; Hirose et al., 1997) and is itself a known risk-factor for peripheral neuropathy (Brizzi and Lyons, 2014). Prior transformation of the neurons or supporting cells of the nerve may therefore be a pre-requisite for target by NK cells.

### Cellular Immunity in Inflammatory Neuropathies

Natural killer cells play an important role in the immune response to infections from EBV, cytomegalovirus (CMV), varicella zoster virus (VZV), and herpes simplex virus (HSV) (Orange, 2013), and each of these can precipitate peripheral neuropathy (Shin and Simpson, 2013; Brizzi and Lyons, 2014). GBS is a rapid-onset autoimmune peripheral neuropathy often preceded by bacterial or viral infection (Hartung, 1999). The most commonly identified prodromal infections are *C. jejuni* (32%), CMV (13%), EBV (10%) and *M. pneumoniae* (5%) (Jacobs et al., 1998). IL-2 is elevated in the serum of GBS patients, with levels returning to normal during the recovery phase (Hartung et al., 1991). However, NK cell activity is lower than baseline within a week of the onset of neuropathic symptoms in GBS (Yoshii and Shinohara, 1998), suggesting exhaustion or loss of NK cell function could exacerbate coincidental neuropathy. Analysis of KIR receptor and human leukocyte antigen (HLA) ligand gene combinations in GBS patients suggests a higher occurrence of inhibitory pairs that would in theory suppress NK cell function (Blum et al., 2014). Thus impairment of an otherwise beneficial innate immune response may contribute to the pathogenesis of GBS. This could explain why treatments designed to suppress the cellular immune response, such as corticosteroids, do not significantly reduce and may actually amplify disease severity (Hughes and van Doorn, 2012).

Chronic inflammatory demyelinating polyneuropathy (CIDP) is an inflammatory neuropathy typified by myelin loss and/or axonal damage of bilateral nerves of the extremities. The disease is characterized by a growing list of auto-antibodies found in patient sera that target peripheral nerve components (Fehmi et al., 2018). CIDP autoantibodies may exert a pathogenic effect by directly interrupting the axonal-myelination structure, or complement-dependent cytotoxicity (Rinaldi and Bennett, 2014). There is also an appreciation of cellular immune component to CIDP (Mathey et al., 2015). Both macrophage phagocytosis (Koike et al., 2018) and clonal expansion of auto-reactive T cells are thought to play a role in the disease, although direct evidence for a myelin antigen-specific auto-reactive T cell population is currently lacking (Schneider-Hohendorf et al., 2012). Reduced cellular cytotoxicity could also be a risk factor in CIDP. For example, missense loss-of-function mutations in perforin have been identified in some CIDP patients (Buttini et al., 2015), and a study of 22 CIDP patients and 22 healthy controls showed the proportion of CD3^–^ CD56^+^ NK cells was lower in the blood of patients (Sanvito et al., 2009). CSF from inflammatory neuropathy patients contains elevated NK cells in association with GBS, whereas CD8^+^ T and CD3^+^ NKT cells levels were increased in CIDP patients (Heming et al., 2019). These findings suggest that heterogeneity in the cytotoxic cellular immune response could play role in differentiating the acute and chronic forms of inflammatory peripheral neuropathy.

## Conclusion

Neurobiologists are increasingly aware of the critical role played by immune function and/or dysfunction in pain syndromes (Hore and Denk, 2019). Despite comprising a relatively minor proportion of peripheral blood cells, NK cells appear to play a unique role in the immune response to peripheral nerve activation and injury, enabled by the remarkable specificity of their innate receptor repertoire and the neuro-immune synapse. One beneficial function that these cells would be particularly suited to is the selective pruning of damaged or aberrantly sprouting peripheral axons following injury or disease. As the archetypal cytotoxic immune cell, NK cells have shone a light on the contribution of other immune cells, including macrophages, neutrophils and CD8^+^ T cells, whose cytotoxic potential in the response to nerve injury is yet to be fully elucidated. A number of chronic pain conditions, as well as pain risk-factors including chronic stress and opioid use, are co-morbid with loss of NK cell function, suggesting NK cell modulation as a potential therapy. Developments in the fields of cancer and immunotherapy are already providing tools to tune NK cells and therefore shift the immune response within the damaged nerve from maladaptive to adaptive. However, the potential role of cellular cytotoxicity in the resolution of neuropathic pain must be weighed against cytotoxic mechanisms themselves precipitating injury and therefore augmenting chronic pain. Thus, maintaining a homeostatic balance – a central tenant of immunology – remains the priority for immune function, including cellular cytotoxicity by NK and other cells, within the peripheral nerve.

## Author Contributions

AD wrote the draft and designed the figures. All authors contributed to writing, editing, and reviewing the manuscript.

## Conflict of Interest

AD, MC, and SO are named on a patent application for the use of immune therapy in the treatment of nerve injury (PCT/KR2019/00806). The remaining author declares that the research was conducted in the absence of any commercial or financial relationships that could be construed as a potential conflict of interest. The handling Editor declared a past co-authorship with one of the authors MC.

## Footnotes

1 https://clinicaltrials.gov/ct2/show/NCT03464799
